# Usefulness of the Nonself-Self Algorithm of HLA Epitope Immunogenicity in the Specificity Analysis of Monospecific Antibodies Induced during Pregnancy

**DOI:** 10.3389/fimmu.2015.00180

**Published:** 2015-05-26

**Authors:** Rene J. Duquesnoy, Marilyn Marrari, Arend Mulder

**Affiliations:** ^1^University of Pittsburgh Medical Center, Pittsburgh, PA, USA; ^2^Leiden University Medical Center, Leiden, Netherlands

**Keywords:** HLAMatchmaker, HLA epitope, nonself–self algorithm, HLA antibody specificity, structural epitope

## Abstract

**Background:**

HLAMatchmaker is a program to analyze the epitope specificities of HLA antibodies. It considers each HLA allele as a string of eplets. Intralocus and interlocus comparisons between donor and recipient alleles offer a structural assessment of compatibility and an analysis of allele panel reactivity patterns can generate information about epitope specificities of HLA antibodies. However, HLAMatchmaker cannot always generate conclusive interpretations of reactivity patterns of all monospecific antibodies, which by definition recognize single epitopes.

**Hypothesis:**

We have therefore developed a new antibody analysis approach that utilizes the nonself–self algorithm of HLA epitope immunogenicity. It is based on the concept that HLA antibodies originate from B-cells with immunoglobulin receptors to self-HLA epitopes on one given allele and which can be activated by epitopes defined by a few nonself residue differences whereas the remainder of the structural epitope of the immunizing allele consists of self residues.

**Methods:**

Three human monoclonal class I antibodies from HLA typed women sensitized during pregnancy were tested in Ig-binding assays with single alleles on a Luminex platform.

**Findings:**

Three new HLA epitopes were identified; they are defined by combinations of nonself- and self-residues for one allele of the antibody producer.

**Conclusion:**

The nonself–self paradigm of HLA epitope immunogenicity offers a second approach to analyze HLA antibody specificities.

## Introduction

The determination of epitope specificities of HLA antibodies offers a powerful approach to assess mismatch acceptability for sensitized recipients. Such epitopes can be described structurally with amino acids in HLA sequence positions. Alleles with epitopes recognized by patient’s antibodies pose an increased risk and may be considered unacceptable mismatches and alleles that lack such epitopes would be acceptable mismatches.

Each HLA allele represents a collection of epitopes with distinct molecular structures defined by immunochemical concepts that address two issues: antigenicity, i.e., the reactivity of epitopes with HLA antibodies and immunogenicity, i.e., the ability of epitopes to induce specific antibodies (1, 2). An understanding of HLA epitope antigenicity must be based on general concepts of how antibody binds to a protein epitope. Three heavy chain and three light chain complementarity determining region loops (CDR-H1, -H2, -H3, -L1, -L2, and -L3) define the binding face (or paratope) of antibody. They interact with a protein epitope that consists of multiple amino acid residues distributed on a molecular surface of 700–900 Å^2^. There are about 15–25 contact residues in what has been referred to as a structural epitope and a centrally located so-called functional epitope consisting of a few residues that bind with CDR-H3, which plays a dominant role in specific binding (3–6).

HLAMatchmaker considers an eplet as the equivalent of a functional epitope and additional residues within a radius of about 15 Å in the corresponding structural epitope are necessary for the binding with antibody (7). Eplets are small configurations of polymorphic amino acid residues generally within a 3-Å radius and they play a dominant role in the specificities of HLA epitopes. Each HLA allele is viewed as a string of eplets and donor–recipient compatibility is assessed through comparisons between donor and recipient eplet strings.

Many studies with informative antibodies have led to a large array of experimentally verified HLA epitopes. The website based International Registry of HLA Epitopes^1^ has listings of antibody-verified epitopes (8–11). One group corresponds solely to a single eplet, i.e., all eplet-carrying alleles react with antibody and the remaining alleles in the panel are non-reactive. The second group of antibody-verified epitopes is defined by eplets that are paired with other amino acid configurations within the structural epitope. Interestingly, such configurations are generally shared between the immunizing allele and at least one allele of the antibody producer (12, 13). This suggests that the alloantibody response to an HLA mismatch has an autoreactive component and recent reports have expanded this view to the so-called nonself–self paradigm of HLA epitope immunogenicity (14, 15).

This paradigm is based on the hypothesis that B-lymphocytes carry low-affinity immunoglobulin B-cell receptors (BCRs) for self-HLA epitopes. Their interactions with self-HLA will not lead to B-cell activation or antibody production. In contrast, exposure to HLA mismatches can induce a strong alloantibody response, which is the result of a productive interaction of a BCR with a nonself eplet whereby the remainder of the structural epitope on the immunizing antigen must be identical or very similar to the corresponding self-HLA epitope of the antibody producer.

HLAMatchmaker has successfully been used to determine epitope specificities of many HLA antibodies especially in conjunction with HLA typing data about antibody producer and immunizer, which provides useful information about the mismatched epitopes presented during the immunizing event (2, 16). This algorithm considers eplets shared between the alleles of the immunizer and antibody producer as intralocus and/or interlocus matches, which cannot elicit antibody responses. Our experience has, however, also shown that HLAMatchmaker can give inconclusive interpretations of the epitope specificities of certain HLA antibodies including human monoclonal antibodies (mAbs), which by definition recognize single epitopes.

We have therefore developed for such antibodies an alternative analysis program, which is based on the nonself–self paradigm of immunogenicity of eplets on immunizing alleles. This report describes the epitope specificity analysis of three human mAbs tested with HLA class I single allele beads (SABs) in antibody binding assays on a Luminex platform.

## Materials and Methods

### Human monoclonal antibodies

Human HLA class I mAbs were obtained from cloned hybridomas generated from Epstein–Barr virus transformed B-cells from women who became sensitized during pregnancy by paternal HLA antigens of their children as previously described (17). These antibodies offer two advantages over sera from sensitized patients: they recognize single epitopes and they are in culture supernatants without interfering factors that can be present in sera. HLA types of antibody producer and immunizer and HLA sequence data provide information about self- and nonself residues of structural epitopes presented during the immunizing event.

### Antibody reactivity assays

These were done using immunoglobulin-binding assays with SAB from two commercial vendors (One Lambda Inc., Canoga Park, CA, USA; Immucor, Life Codes Corporation, Stamford, CT, USA) according to manufacturer’s instructions. In brief, an aliquot of an SAB mixture was incubated with a small volume of antibody, then washed to remove unbound antibody. Anti-human immunoglobulin (IgG or IgM) antibody conjugated to phycoerythrin was added; after incubation, the bead mixture was diluted for analysis on a Luminex 100 instrument (Luminex, Austin, TX, USA) and the reactivity was determined with the manufacturer’s software. Median fluorescence intensity (MFI) values were recorded for each allele and the positive and negative control beads. All mAbs showed extremely low MFI values (mostly <100) with the self-alleles of the antibody producer.

### Design of the antibody reactivity analysis program based on the nonself–self paradigm of HLA epitope immunogenicity

This paradigm considers the nonself–self theory of the immune response originally forwarded by Burnet (18) and extensively discussed and modified by many investigators (19–23). During B-cell development, rearrangements of V_H_ and V_L_ genes produce diversity in the antigen-binding sites of immunoglobulins. These processes lead to the expression of immunoglobulin receptors on developing B-cells, which go through several stages to become mature lymphocytes with BCRs that can recognize epitopes on autologous proteins. Lymphocytes with high-avidity BCRs disappear after positive and negative selection and receptor editing and the remaining B-cells carry only low-avidity BCRs so that their interactions with self-epitopes on autologous proteins will not induce their activation. B-cell responses leading to antibodies cannot be triggered by every foreign entity, but it requires a “criterion of continuity” of antigenic patterns, which discriminate nonself from self (22). In other words, the immune system does not react to self-molecules, but will respond to certain modifications within self-molecules.

We have previously hypothesized that B-cells carry low-affinity receptors for self-HLA epitopes, which can interact with nonself residue configurations provided that the remainder of the structural epitope of the immunizing HLA antigen consists of self-residues (1, 14). It is well-known that among the six CDRs immunoglobulins, the centrally located CDR-H3 binds to a functional epitope and plays a pivotal role in determining antibody specificity. CDR-H3 has a considerably longer amino acid sequence than the other CDRs and displays significant sequence variability and structure (24, 25). According to our hypothesis, CDR-H3 has a loop configuration with very low binding ability with a self-HLA functional epitope but it can refold to yield another configuration that can specifically recognize a nonself functional HLA epitope whereas the other CDRs bind to self-configurations shared between the immunizing antigen and an allele of the antibody producer. This concept raises the requirement that the immunizing HLA antigen must have one distinct nonself amino acid configuration (the mismatched eplet), whereas the other amino residues contacted by antibody should be the same or very similar as those on a self-HLA antigen of the antibody producer. Three publications describe experimental evidence, which supports our hypothesis (1, 14, 15). For each antibody response, at least one allele of the antibody producer has no or few differences with the immunizing allele in antibody-accessible positions defined by a 15 Å radius of the mismatched eplet, the presumed dimension of a structural epitope.

This antibody reactivity analysis has three steps in the specificity determination of monospecific antibodies. Step 1: identify from amino acid sequences, which residues (not eplets) on the molecular surface of the immunizing allele are nonself for at least one HLA-A, HLA-B, or HLA-C allele of the antibody producer but are self for other alleles of the antibody producer. This step applies the concept that the antibody has originated from a B-cell with a BCR specific for a self-epitope on one of the HLA alleles of the antibody producer. Step 2: select nonself residue(s) of the immunizing allele, which are present on all antibody-reactive alleles in the SAB panel; none of the reactive alleles can have a different residue in that sequence position. This step applies the concept that a monospecific antibody recognizes an epitope defined by the selected nonself residue presented by the immunizing allele. Some non-reactive SAB alleles including those of the antibody producer will have the same nonself residue but within the corresponding structural epitope, they have certain residue configurations that interfere with antibody binding. Step 3: within a 15 Å radius, compare the residue compositions of nonself-carrying reactive and non-reactive alleles including those of the antibody producer. These comparisons are designed to identify residues associated with a lack of binding with antibody. They permit molecular descriptions of antibody-specific epitopes defined by nonself residues combined with distinct configurations of self-residues within the context of structural epitopes.

### Structural modeling of epitopes

Residue locations defining epitopes on the HLA molecular surface were visualized with crystallographic models downloaded from the Entrez Molecular Modeling Database (MMDB) on the National Center for Biotechnology Information website^2^. This was done with the Cn3D structure and sequence alignment software program, which can show selected residues and has a “select by distance” (in Ångstroms) command that allows the user to determine how far residues are apart. (26).

## Results

Tables 1–3 show the MFI values of the SAB panel and the amino acid residues used to determine the epitope specificities of the mAbs. As a visual tool for this analysis, these residues are color coded. Nonself residues presented by the immunizing alleles are in yellow boxes and self-residues that are shared between the immunizer and the alleles of the antibody producer are in blue boxes. Residues associated with negative reactions of panel alleles are in orange red boxes. Some reactive alleles have in certain sequence positions different residues than the immunizing allele; such permissive residue substitutions are in green boxes.

**Table 1:** **HU-62 is specific for an epitope defined by 142I145R + 138M + 79G80T82R83G**.

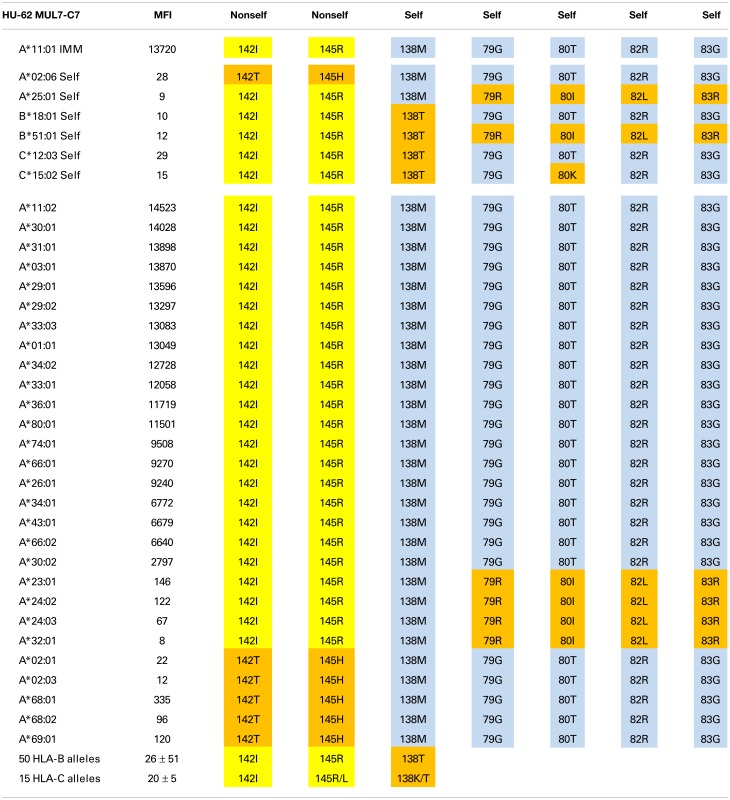

### An epitope defined by 142I145R + 138M + 79G80T82R83G

**Figure 1:**
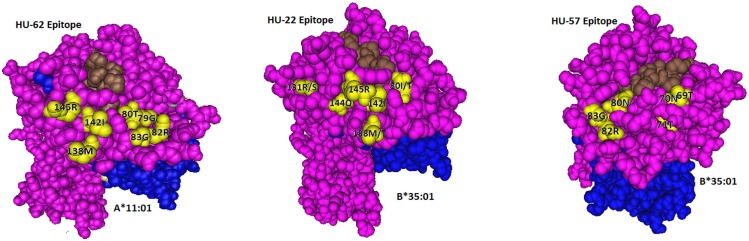
**Locations of residues (in yellow) defining the HU-62, HU-22, and HU-57 epitopes**. The HLA-bound peptide is in brown and β2-microglobulin is in blue.

The A*11:01-induced HU-62 mAb (MUL7-C7, IgM) reacted with 20 HLA-A alleles MFI values ranging from 14,523 to 2,797 (Table 1). The immunizing A*11:01 (indicated in the table as IMM) has 142I and 145R that are shared with the antibody-reactive alleles. The 142I145R combination is mismatched for A*02:06, which has 142T145H but the other alleles of the antibody producer have 142I145R. According to the nonself–self algorithm of HLA epitope immunogenicity, HU-62 antibodies must have originated from B-cells with BCRs toward a 142T145H-defined self-epitope on HLA-A*02:06 and which responded to the nonself 142I145R on the immunizing A*11:01. However, why does HU-62 not react with so many 142I145R-carrying alleles including those of the antibody producer? An analysis of residues within 15 Å of 142I145R indicated an important role of sequence position 138. The immunizing A*11:01 and all reactive HLA-A alleles have a methionine (138M) residue. In contrast, the non-reactive HLA-B and HLA-C alleles all of which have 142I145R, carry either a threonine (138T) or a lysine (138K) residue; they include B*18:01, B*51:01, and C*12:03 of the antibody producer. This suggests that 138M is a required component of the 142I145R-defined epitope recognized by HU-62. However, the 142I145R + 138M-carrying A*25:01 of the antibody producer and four more alleles in the SAB panel (A*23:01, A*24:02, A*24:03, and A*32:01) were non-reactive. They share the 79G80I82L83R combination, whereas the reactive alleles have 79G80T82R83G. These findings suggest that HU-62 is specific for an epitope defined by the combination of 142I145R + 138M + 79G80T82R83G. Figure 1 (left) shows the locations of these residues on a molecular model of A*11:01; all of them are <10 Å from each other.

Not surprisingly, the 142T145H-carrying A*02, A*68, and A*69 alleles were non-reactive although all of them have 138M + 79G80T82R83G. It should be noted that these residues are shared between the immunizing A*11:01 and A*02:01 of the antibody producer. These findings suggest that the HU-62 antibody response was initiated by an antigen with a small residue difference (142I145R versus 142T145H) whereas the rest of the epitope consisted of self-residues.

### An epitope defined by 142I144Q145R + 80I or 80T

The A*32:01-induced mAb HU-22 (HDG2-G7, IgG) reacted with 16 additional HLA-A alleles and 17 HLA-B alleles; their MFI values ranged from 21,369 to 3,788 (Table 2). An analysis of the nonself residues shared between the immunizing allele and the antibody-reactive alleles showed that 142I, 144Q, and 145R are nonself for the antibody producer’s A*02:01, which has 142T,144K, and 145H whereas 144Q alone is nonself for the antibody producer’s A*24:02, which has 144K. This suggests that HU-22 recognizes an epitope that might be structurally similar to the HU-62 epitope, which is defined by 142I145R + 138M + 79G80T82R83G. HU-22, however, reacted with both HLA-A and HLA-B alleles and this rules out 138M as a critical component of this epitope. Another difference is that the HU-22 epitope requires 142I144Q145R as indicated by the non-reactive B*13 alleles, which have 142I144Q145L and the non-reactivity of all seven 142I144K145R-carrying HLA-A alleles (bottom of Table 2). Sequence position 80 appears to play an important role in the HU-22 epitope. The immunizing A*32:01 has 80I and the reactive alleles have either 80I or 80T, which suggests that 80T is a permissible residue substitution that does not significantly affect the reactivity with HU-22. In contrast, all 29 non-reactive 142I144Q145R-carrying HLA-B alleles have 80N and all HLA-C alleles, which have 142I144Q145R with either 80N or 80K are non-reactive thereby suggesting that these residues prevent binding with HU-22.

**Table 2:** **HU-22 is specific for an epitope defined by 142I144Q145R + 80I or 80T**.

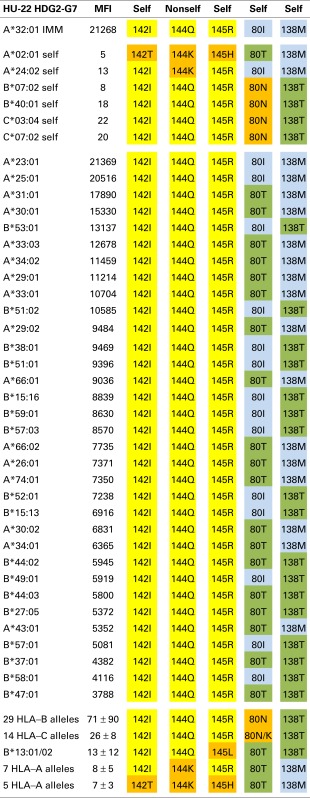

*Self- and nonself residue assignments are based on A*24:02 of the antibody producer.

As noted above, the antibody-reactive alleles showed a wide range of MFI values from 21,369 to 3,788. Does this reflect residue differences within the structural epitope that corresponds to 142I144Q145R? The average MFI values for 80I and 80T-carrying reactive alleles were similar (9108 ± 5778 versus 8122 ± 3781, *p* = 0.24 by Student’s *t*-test). On the other hand, alleles with 138M (which was presented by the immunizing A*11:01) were significantly more reactive than the 142I144Q145R-carrying alleles that have 138T (10678 ± 5778 versus 7146 ± 2567, *p* = 0.008). Within the 138M or 138T groups, there were also differences between 80I (which was presented by the immunizing A*11:01) and 80T namely 21051 ± 466 versus 9914 ± 3584, *p* < 0.0001 and 8361 ± 2486 versus 4835 ± 936, *p* = 0.0008, respectively. These findings show that these residue differences decrease the MFI values but not to an extent that they reflect negative reactions.

In conclusion, HU-22 is specific for an epitope defined by 142I144Q145R combined with 80I or 80T. This epitope is depicted in Figure 1 (middle). According to the nonself–self paradigm of HLA epitope immunogenicity, it seems likely that HU-22 originated from B-cells with BCRs specific for a self-epitope on A*24:02 of the antibody producer and the immunizing A*32:01 had the nonself 144Q together with the self 142I, 145R, and 80I residues. Another explanation is that HU-22 might have been produced by B-cells with BCRs specific for a self-epitope on A*02:01. In such case, the immunizing A*32:01 presented a nonself 142I144Q145R together with a separate nonself 80I residue that would have made contact with a different CDR of antibody.

### An epitope defined by 69T70N71T + 80N82R83G

The B*15:01-induced HU-57 (DMS4G2, IgG) reacted only with alleles that share the combination of the 69T70N71T and 80N82R83G configurations; all other alleles lack this combination and are essentially non-reactive (Table 3). It should be noted that B*51:01 of the antibody producer has 69TNT whereas B*07:02 and C*07:02 have 80NRG. From which autoreactive B-cells did HU-57 originate? There are two possibilities. First, the antibody producer had B-cells with BCRs specific for a 69A70Q71A-defined self-epitope on B*07:02 and 69T70N71T on the immunizing B*15:01 was recognized as nonself whereas 80N82R83G is self. Second, HU-57 could have originated from B-cells with BCRs for the 80I82L83R-defined self-epitope on B*51:01 of the antibody producer and the nonself 80N82R83G on the immunizing B*15:01-induced activation. All 10 HLA-B alleles with 69T70N71T but not 80N82R83G and all 9 HLA-B alleles with 80N82R83G but not 69T70N71T were considered non-reactive but we noted slightly higher MFI values for the first group: 477 ± 178 versus 295 ± 55, *p* = 0.01 (*t*-test). This finding seems to favor the possibility that HU-57 originated from B*07:02 autoreactive B-cells. Figure 1 (right) shows the molecular locations of the residues that define the HU-57 epitope on B*35:01.

**Table 3:** **HU-57 is specific for an epitope defined by 69T70N71T + 80N82R83G**.

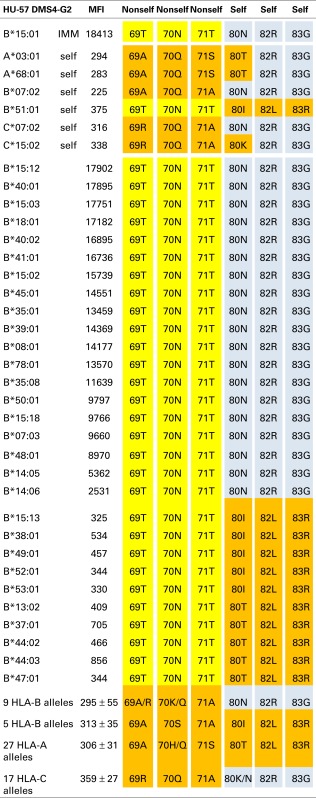

### Polymorphic residue comparisons between structural alloepitopes and self-epitopes

The nonself–self algorithm of HLA epitope immunogenicity mandates that an immunizing epitope has a nonself residue component, which in the context of the structural epitope is surrounded by a residue configuration that is the same or very similar to that of a self-epitope on an HLA allele of the antibody producer. The 15 Å radius is an estimate of the dimension of a structural epitope (7). Table 4 shows the polymorphic residues on the molecular surface within 15 Å of nonself configurations of the epitopes recognized by each of these three mAbs in comparison with the corresponding self-epitope on an HLA allele of the antibody producer who presumably had B-cells with the appropriate self-BCRs.

**Table 4:** **Polymorphic residue comparisons between mAb defined and corresponding self-HLA structural epitopes**.

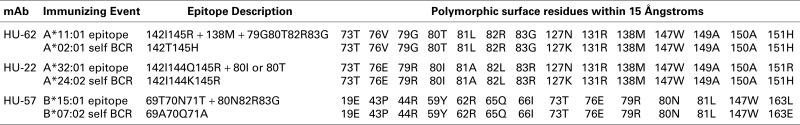

For the A*11:01-induced epitope recognized by HU-62, position 127 is the only one among 14 polymorphic residue positions with a difference with the antibody producer’s A*02:01 namely, 127N versus 127K. The SAB panel had no informative alleles, which could have assessed the influence of 127N and 127K on the epitope recognized by HU-62. For the HU-22 epitope, the immunizing A*32:01 and the antibody producer’s A*24:02 have differences in two positions: 127N versus 127K and 151R versus 151H. Since neither residue difference had any significant influence on the HU-22 reactivity with the SAB panel (data not shown), it seems unlikely that they influence the structural epitope recognized by HU-22. For the B*15:01-induced HU-57 epitope, there is only one position with a residue difference: 163L versus 163E. Alleles with different residues in position 163 were equally reactive with HU-57 (data not shown) thereby suggesting no significant role in the epitope recognized by HU-57.

It should be noted that there also are monomorphic residues within the 15 Å radius of a nonself residue configuration (data not shown); such residues are always self. Consistent with the nonself–self algorithm of HLA epitope immunogenicity, these findings indicate a very high degree of selfness of the residues surrounding a nonself residue configuration of an immunizing HLA epitope.

## Discussion

This report describes the epitope specificities of three human mAbs determined with a new approach that is based on the nonself–self paradigm of HLA epitope immunogenicity. Although HLAMatchmaker is generally considered an effective program for the epitope specificity analysis of HLA antibodies, our experience has shown that for certain mAbs it does not permit a precise specificity determination. HLAMatchmaker considers each allele as a string of eplets covering the entire sequence of polymorphic residues and matching is done by intralocus and interlocus comparisons between donor and recipient strings. A donor eplet that is also present on any of the recipient alleles is considered a match and according to HLAMatchmaker cannot induce antibodies.

There are, however, exceptions, which can be explained with the hypothesis that each HLA antibody population originates from an immature B-cell with BCRs specific for a given structural self-epitope defined by residues of an HLA allele of the recipient. Each recipient has a repertoire of B-cells with BCRs for various self-HLA epitopes. A donor allele can activate certain autoreactive B-cells provided it presents an epitope with some distinct residue differences but is otherwise structurally similar to the recipient’s self-epitope. In other words, an immunizing HLA epitope must have one or few nonself residues surrounded by self residues in context with the corresponding structural self-epitope of the antibody producer. Previous reports of HLAMatchmaker identified epitopes have indeed shown that within a 15 Å radius antibody-reactive eplets are surrounded by residues shared with at least one of the alleles of the antibody producer (1, 14, 15).

This report gives examples of the situation whereby the nonself residue part of the immunizing epitope can be found on one allele but not the other alleles of the antibody producer. For HU-62, for instance, the immunizing A*11:01 presents 142I145R as nonself for A*02:06 of the antibody producer but this configuration is also present on her HLA-B and HLA-C alleles (Table 1). According to HLAMatchmaker, 142I145R would be an interlocus match. HU-57 is another example: B*15:01 presents 69T70N71T as a mismatch for B*07:02 but this configuration would be considered as an intralocus match for B*51:01 of the antibody producer (Table 3). This enigma can be resolved by considering the data that these epitopes are defined by the overall combinations of nonself and self residue configurations.

Accordingly, HU-62 is specific for an epitope defined by the nonself 142I145R in combination with two self-configurations: 138M and 79G80T82R83G (Table 1) and HU-57 recognizes an epitope defined by nonself 69T70N71T + self 80N82R83G (Table 3). Interestingly, HU-22 is specific for an epitope that is defined by nonself 142I144Q144R combined with self 80I and self 138M (for the antibody producer’s A*24:02) but 80T and 138T are permissible substitutions that have only a minor effect on the binding with antibody (Table 2).

It should be noted that in all three tables, the reactive alleles in the panel showed wide ranges of MFI values. Certain alleles may have other structural differences with the immunizing allele, which cause lower MFI values but which should still be considered as positive. A discussion of these concepts is beyond the scope of this paper.

Altogether, this study has yielded three new epitopes, which can only be defined by combinations of nonself and self residues for one allele of the antibody producer. They will be added to the list of antibody-verified HLA class I epitopes in the International HLA epitope registry. Each epitope has a distinct residue description that will be converted to eplet combinations, which can be used in the HLAMatchmaker analysis of epitope specificities of HLA antibodies.

The nonself–self paradigm of HLA epitope immunogenicity offers a new alternative approach to investigate the specificities of antibodies with complex reactivity patterns. It may also offer opportunities to study antibody responses to HLA mismatches in a transplant setting.

## Conflict of Interest Statement

The authors declare that the research was conducted in the absence of any commercial or financial relationships that could be construed as a potential conflict of interest.

## Footnotes

^1^http://www.epregistry.com.br

^2^http://www.ncbi.nlm.nih.gov/Structure
